# Zebrafish (*Danio rerio*) physiological and behavioural responses to insect-based diets: a multidisciplinary approach

**DOI:** 10.1038/s41598-020-67740-w

**Published:** 2020-06-30

**Authors:** Matteo Zarantoniello, Basilio Randazzo, Giorgia Gioacchini, Cristina Truzzi, Elisabetta Giorgini, Paola Riolo, Giorgia Gioia, Cristiano Bertolucci, Andrea Osimani, Gloriana Cardinaletti, Tyrone Lucon-Xiccato, Vesna Milanović, Anna Annibaldi, Francesca Tulli, Valentina Notarstefano, Sara Ruschioni, Francesca Clementi, Ike Olivotto

**Affiliations:** 1 Dipartimento di Scienze della Vita e dell’Ambiente, Università Politecnica delle Marche, via Brecce Bianche, 60131 Ancona, Italy; 2 Dipartimento di Scienze Agrarie, Alimentari ed Ambientali, Università Politecnica delle Marche, via Brecce Bianche, 60131 Ancona, Italy; 3 Dipartimento di Scienze della Vita e Biotecnologie, Università di Ferrara, via L. Borsari 46, 44121 Ferrara, Italy; 4 Dipartimento di Scienze Agro-Alimentari, Ambientali e Animali (Di4A), Università di Udine, via Sondrio 2/A, 33100 Udine, Italy

**Keywords:** Metabolism, Fat metabolism, Animal physiology

## Abstract

Black Soldier Fly (BSF) meal is considered as an alternative, emerging and sustainable ingredient for aquafeed production. However, results on fish physiological responses are still fragmentary and often controversial, while no studies are available on fish behavior in response to these new diets. The present work represents the first comprehensive multidisciplinary study aimed to investigate zebrafish physiological and behavioural responses to BSF-based diets. Five experimental diets characterized by increasing inclusion levels (0, 25, 50, 75 and 100% respect to fish meal) of full fat BSF prepupae meal were tested during a 2-months feeding trial. Prepupae were cultured on coffee silverskin growth substrate enriched with a 10% *Schizochytrium* sp. to improve insects’ fatty acids profile. The responses of zebrafish were assayed through biometric, histological, gas chromatographic, microbiological, spectroscopic, molecular and behavioural analyses. Results evidenced that BSF-based diets affected fish fatty acid composition, while behavioural tests did not show differences among groups. Specifically, a 50% BSF inclusion level diet represented the best compromise between ingredient sustainability and proper fish growth and welfare. Fish fed with higher BSF inclusions (75 and 100%) showed hepatic steatosis, microbiota modification, higher lipid content, fatty acid modification and higher expression of immune response markers.

## Introduction

The promotion of high-quality fish production as well as fish welfare are the main aquaculture goals, both strictly related to an adequate fish nutrition^1^. The use of fish meal (FM) and fish oil (FO) in aquafeed formulation is no more feasible because of important environmental and economic issues^2^. Over the last decades, several alternative ingredients (plant origin proteins, microalgae and processed animal proteins) to FM and FO have been tested^3^. However, each of these ingredients showed some disadvantages in its application in aquafeed formulation including unbalanced amino acid profile, poor protein digestibility, presence of anti-nutritional factors and high production costs^3,4^.

Insects are now considered as an alternative and sustainable ingredient for feed production^5^. In particular, the Black Soldier Fly (*Hermetia illucens*; BSF) larvae are one of the most promising candidates because of their proper protein content and the amino acid composition similar to that of FM^6,7^. In addition, BSF have low environmental requirements, a high feed conversion efficiency and they can growth on organic by-products, promoting sustainability and the circular economy concept in the aquaculture sector^8,9^. Several studies tested different BSF inclusion levels in aquafeed formulation but results on fish physiological responses are still controversial, while behavioural effects on fish are completely missing^10–13^. The use of BSF in aquafeed has been shown, at certain inclusion levels, to improve gut health, immunity and general fish welfare^14^. As an example, BSF contains lauric acid and chitin, which showed, at certain concentrations, anti-inflammatory and immune-boosting properties^14,15^. Additionally, BSF-based diets have been proven to increase biodiversity in fish microbiome community structure^16–18^, which in turn, has an important role in host metabolism, nutrition, immunity and welfare^18,19^.

However, the use of insect meal in aquafeed formulation still faces some bottlenecks^5^. Most insect species, including BSF, are rich in saturated fatty acids (SFA) and contain negligible amounts of polyunsaturated (PUFA) ones^20^. A high dietary SFA content, coupled with a high n6/n3 ratio, has been shown to play a key role in fish hepatic steatosis development^13,21^. Furthermore, insect meal may alter the biochemical characteristics of fish fillet, especially regarding the fatty acid composition, even in absence of major alterations of its sensory properties^12,22^.

While a diet rich in n3 fatty acids has generally been associated with a lower risk to develop neurodegenerative and cardiovascular diseases in humans and animal models^23,24^, the chronic ingestion of n6 and SFAs has been related to wide loss of brain volume and synapses^25^ and to behavioural impairments such as dementia^26^. Impaired behaviour and learning have also been related to a reduction in gut microbiota variability^27^, a condition often related to a n6 and SFAs rich diet^28^.

Given their unbalanced FAs profile, BSF-based diets could have direct effect on fish welfare and neuronal functioning. At this regard, BSF larvae fatty acid composition can be modified by the growth substrate^6^. Specifically, Truzzi et al. (2020)^9^ and Zarantoniello et al. (2020)^19^ demonstrated that culturing BSF larvae on an organic substrate enriched with 10% *Schizochytrium* sp*.* significantly increased insects’ PUFAs content. With respect to previous studies carried out on zebrafish^21,29^, this enriching procedure allows to include up to 50% of BSF meal in the fish diet without impairing fish physiology, and hence represents a remarkable example of sustainable circular economy. Zebrafish represents an ideal organism to better understand fish physiological responses to new ingredients^30^ and an emerging useful model organism for neuroscience/behavioural research^31^.

The present work represents the first comprehensive multidisciplinary study aimed to investigate zebrafish physiological and behavioural responses to BSF-based diets. Five experimental diets characterized by increasing inclusion levels of full-fat BSF prepupae meal with respect to FM were tested during a two-months feeding trial. BSF prepupae were cultured on a coffee silverskin growth substrate enriched with a 10% *Schizochytrium* sp. to improve insects’ FAs profile^9^.

The physiological responses of zebrafish were analysed through biometric, histological, gas chromatographic, microbiological, spectroscopic and molecular analyses. Behaviour was assessed with an open-field exploration test and a standard photic entrainment test.

## Results

### Growth and survival

**Figure 1:**
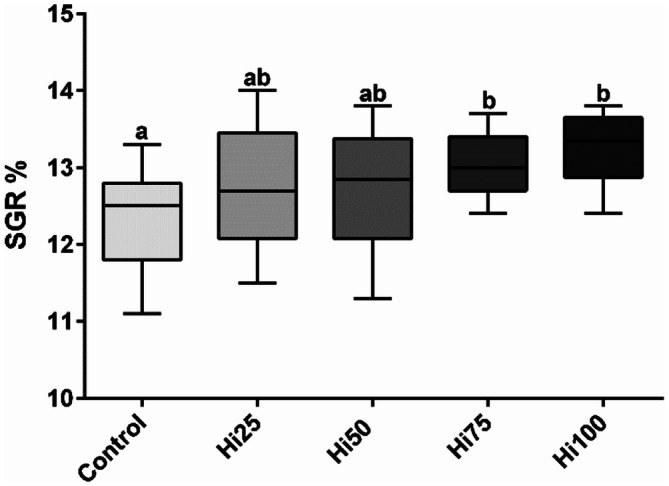
Zebrafish fed experimental diets specific growth rate (% weight growth day^−1^). Zebrafish fed diets including 0, 25, 50, 75 and 100% of BSF meal (Control, Hi25, Hi50, Hi75 and Hi100, respectively). Boxplots show minimum and maximum (whiskers), first quartile, median and third quartile (box). Different letters indicate statistically significant differences among experimental groups (*p* < 0.05).

Considering specific growth rate (Fig. 1), only Hi75 and Hi100 groups (13.0 ± 0.4 and 13.2 ± 0.4%, respectively) showed significantly (*p* < 0.05) higher values than Control (12.3 ± 0.6%). No significant differences were evident among Hi25, Hi50 and Control group (12.7 ± 0.8 and 12.7 ± 0.9% for Hi25 and Hi50, respectively). As regards survival, no significant differences were observed among the experimental groups.

### Fatty acid content and composition

#### Diets

**Figure 2:**
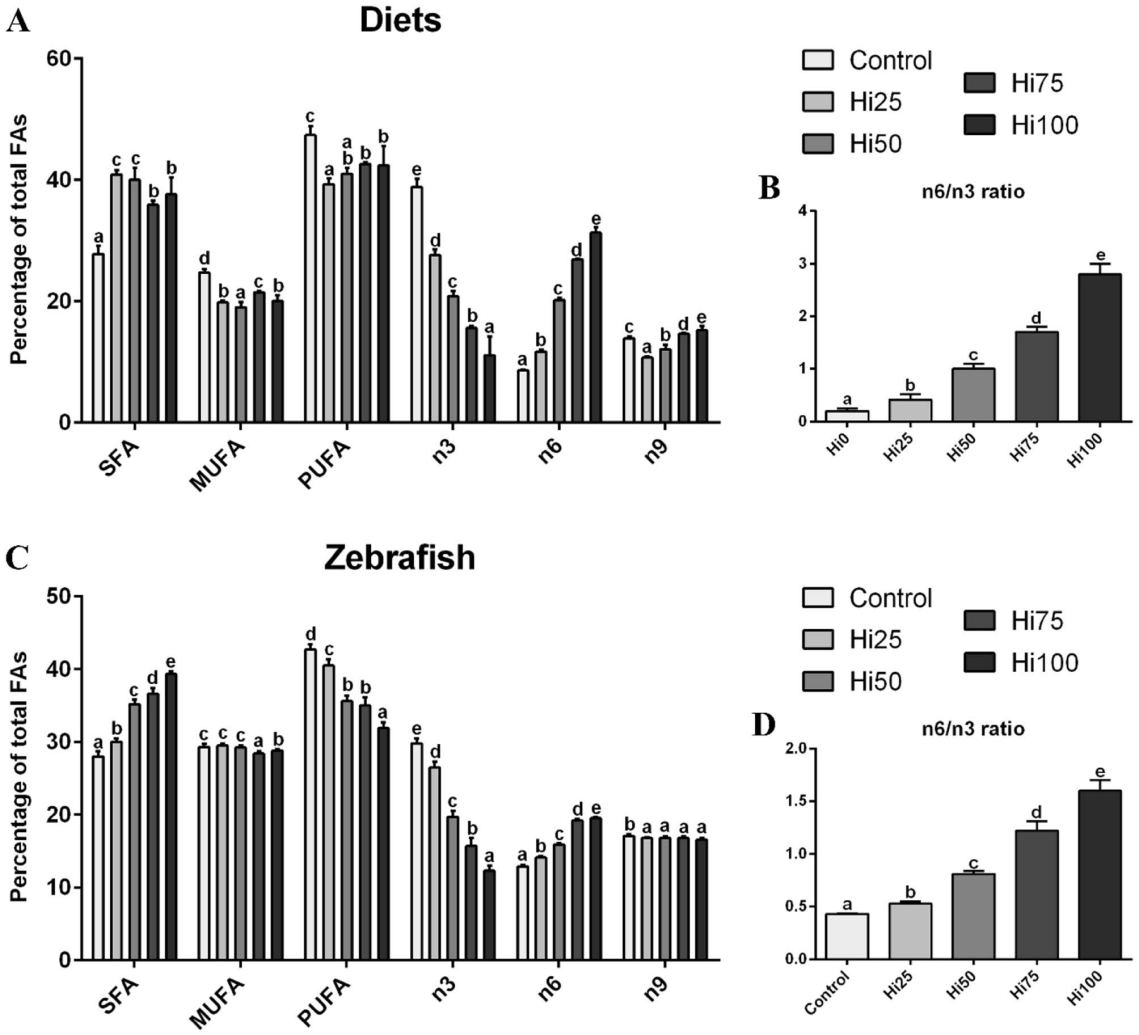
Content of SFA, MUFA and PUFA fatty acid (as % of total FA) and omega 3 (n3), omega 6 (n6) and omega 9 (n9) fatty acid contribution to lipid profile. (**A**,**B**) experimental diets including increasing BSF meal percentages (0, 25, 50, 75, and 100%, respect to fish meal) and (**C**,**D**) zebrafish fed on the different diets. Different letters show statistically significant differences among experimental groups compared within the same fatty acid class (*p* < 0.05). Values are showed as mean ± SD (n = 3 for experimental diets; n = 15 for zebrafish).

Results obtained from the experimental diets were previously reported in Zarantoniello et al. (2020)^19^. Briefly, as reported in Fig. 2A, BSF-based diets showed significantly (*p* < 0.05) higher percentages of SFA and significantly (*p* < 0.05) lower percentages of MUFAs and PUFAs with respect to Control diet. However, increasing BSF full-fat prepupae meal inclusion levels in the diets resulted in a PUFAs increase from Hi25 to Hi100. Increasing inclusion levels of BSF full-fat prepupae meal in the diets resulted in a significant decrease (*p* < 0.05) of n3 percentages and a parallel significant (*p* < 0.05) increase in n6 percentages. Consequently, the n6/n3 ratio showed significant differences (*p* < 0.05) among experimental diets, increasing from Control to Hi100 diets (Fig. 2B).

#### Zebrafish

Figure 2C reports the FAs classes percentages of zebrafish fed the different diets. The increasing inclusion levels of enriched BSF prepupae meal resulted in: a significant (*p* < 0.05) increase in SFA (28.0 ± 0.7, 30.0 ± 0.5, 35.2 ± 0.6, 36.6 ± 0.8 and 39.2 ± 0.4% for Control, Hi25, Hi50, Hi75 and Hi100, respectively) and n6 (12.9 ± 0.2, 14.1 ± 0.2, 15.9 ± 0.2, 19.2 ± 0.2 and 19.5 ± 0.2% for Control, Hi25, Hi50, Hi75 and Hi100, respectively) percentages; a significant (*p* < 0.05) decrease in both PUFA (42.7 ± 0.7, 40.5 ± 0.8, 35.6 ± 0.8, 35.0 ± 1.1 and 31.9 ± 0.8% for Control, Hi25, Hi50, Hi75 and Hi100, respectively) and n3 (29.3 ± 0.7, 26.5 ± 0.8, 19.7 ± 0.8, 15.7 ± 1.1 and 12.3 ± 0.7% for Control, Hi25, Hi50, Hi75 and Hi100, respectively) percentages. Regarding monounsaturated fatty acids (MUFA) content, groups fed with the higher BSF inclusion levels showed significantly (*p* < 0.05) lower percentages (28.4 ± 0.3 and 28.8 ± 0.2% for Hi75 and Hi100, respectively) with respect to Control; Control, Hi25 and Hi50 (29.3 ± 0.4, 29.5 ± 0.3 and 29.2 ± 0.3, respectively) did not show significant differences among them. Considering n9 content, all the groups fed BSF-based diets were characterized by significantly (*p* < 0.05) lower percentages (16.8 ± 0.1, 16.8 ± 0.2, 16.8 ± 0.2 and 16.6 ± 0.2% for Hi25, Hi50, Hi75 and Hi00, respectively) than Control (17.1 ± 0.2%). Finally, the n6/n3 ratio (Fig. 2D) increased according to the increasing dietary BSF meal inclusion (0.43 ± 0.01, 0.53 ± 0.02, 0.81 ± 0.03, 1.22 ± 0.09 and 1.60 ± 0.10 for Control, Hi25, Hi50, Hi75 and Hi100, respectively).

As concern zebrafish FAs composition (for details please see Supplementary Table S2), the most represented SFAs in all the experimental groups were palmitic acid (16:0) and stearic acid (18:0). Furthermore, the percentage of major (> 1%) saturated fatty acids (18:0 excluded) significantly (*p* < 0.05) increased according to the increasing BSF meal inclusion. In particular, the content of lauric acid increased up to ~ 30-folds from Control to Hi100 group. Considering MUFAs, the predominant fatty acid in all the experimental groups was oleic acid (18:1n9) which did not show significant differences among the experimental groups. Furthermore, the other major MUFAs such as 16:1n7 and 18:1n7 showed a significant decrease in fish fed diets with increasing BSF meal inclusion levels from 50% (Hi50) to 100% (Hi100). Linoleic (18:2n6) and docosahexaenoic (22:6n3; DHA) acids were the most abundant PUFAs in all the dietary treatments. Linoleic acid and arachidonic acid (20:4n6) showed a significant (*p* < 0.05) increase in fish fed diets with increasing BSF meal inclusion levels. A significant (*p* < 0.05) decrease in eicosapentaenoic acid (20:5n3; EPA) percentage was detected in fish fed diets with increasing BSF meal inclusion levels, whereas, as regards DHA, a similar, but milder trend was observed respect to EPA. Consequently, the DHA/EPA ratio significantly increased with the increasing BSF meal inclusion levels in the diets.

### Histology

**Figure 3:**
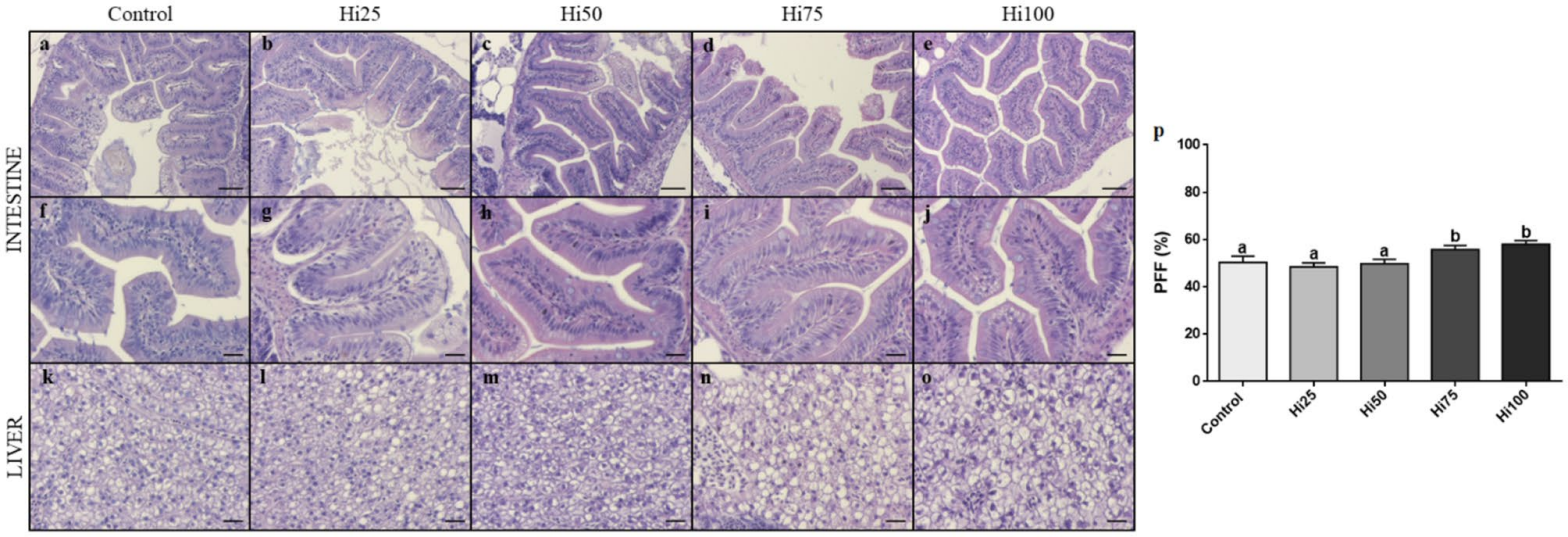
Zebrafish (Control, Hi25,50,75,100 groups) intestine and liver histology and percentage of fat fraction (PFF) in liver tissue. **a–j**: intestine; **k–o**: liver; **p**: PFF%. Histology scale bars: **a–e**:50 µm; **f–j**: 20 µm; **k–o**: 10 µm. For PFF%, values are showed as mean ± SD (n = 15). Different letters indicate statistically differences among the experimental groups (*p* < 0.05).

Histological analyses were performed in order to detect possible inflammatory events in fish intestine and to evaluate lipid accumulation or steatosis in liver. As concerns intestine (Fig. 3a–j), no morphological alteration or sign of inflammation was evident in all the experimental groups (Fig. 3a,f: Control; Fig. 3b–e and g–j: Hi groups).

Considering liver, results evidenced a variable degree of lipid accumulation in all the experimental groups (Fig. 3k–o). Control, Hi25 and Hi50 groups were characterized by a modest fat liver parenchima with a diffuse presence of hepatocytes with cytoplasm filled of fat, interspersed with normal hepatocytes. Conversely, Hi75 and Hi100 groups showed a severe degree of steatosis with swollen hepatocytes and abundant intracytoplasmic lipid accumulation (Fig. 3n,o). These results were confirmed by the statistical quantification of the fat percentage fraction (PFF%; Fig. 3p) on liver sections. In particular, no significant differences were evident among Control, Hi25 and Hi50 groups (50.3 ± 2.6, 48.3 ± 1.7, 50.0 ± 2.0% for Control, Hi25 and Hi50, respectively), while Hi75 and Hi100 groups (55.6 ± 1.7 and 58.0 ± 1.4% for Hi75 and Hi100, respectively) showed significantly (*p* < 0.05) higher values respect to the other experimental groups.

### FTIR analysis

#### Brain samples

**Figure 4:**
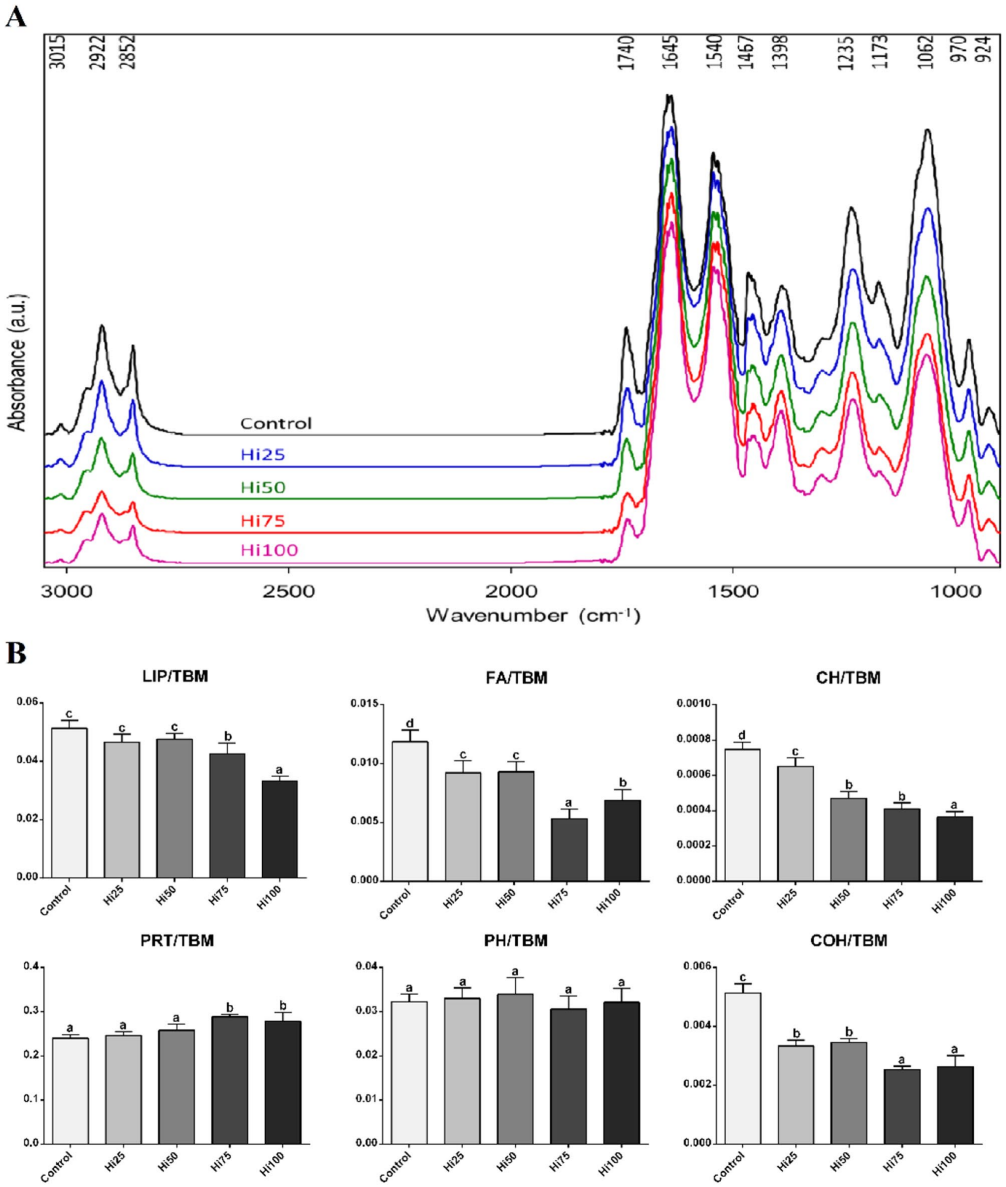
(**A**) Average absorbance spectra and (**B**) biochemical composition of Zebrafish brain samples of all dietary groups. (**A**) Spectra are reported in the 3050–900 cm^-1^ spectral range and are shifted along the y-axis for a better understanding. The wavenumbers of the most significant bands are reported in the upper part. (**B**) Univariate analysis of the following band area ratios: LIP/TBM (relative amount of total lipids); FA/TBM (relative amount of fatty acids); CH/TBM (degree of unsaturation in lipid alkyl chains); PRT/TBM (relative amount of total proteins); PH/TBM (relative amount of phosphate groups), and COH/TBM (relative amount of carbohydrates). Data are reported as mean ± SD. Statistically significant differences are indicated by different letters (*p* < 0.05). Zebrafish fed diets including 0, 25, 50, 75 and 100% of BSF meal (Control, Hi25, Hi50, Hi75 and Hi100, respectively).

The average spectra of brain samples of all dietary groups are reported in Fig. 4a. The most significant IR bands (reported in the upper part of Fig. 4a), are listed below, together with the position (wavenumbers) and the biological meaning: ~ 3015 cm^-1^ (=CH moieties in lipid alkyl chains); ~ 2922 cm^−1^ and ~ 2852 cm^−1^ (CH_2_ groups in lipid alkyl chains); ~ 1744 cm^−1^ (C=O moiety in lipids and fatty acids); ~ 1645 cm^−1^ and ~ 1540 cm^−1^ (Amide I and II bands of proteins, respectively); ~ 1467 cm^−1^ (CH_2_ and CH_3_ groups in lipids and proteins’ side chains); ~ 1398 cm^−1^ (COO- groups in amino acids); ~ 1235 cm^−1^ (phosphate groups); ~ 1173 cm^−1^ (glycosylated compounds); ~ 1062 cm^−1^ (carbohydrates); ~ 970 cm^−1^ and ~ 925 cm^−1^ (A-DNA and Z-DNA, respectively).

The univariate analysis of the biochemical composition of brain samples of all dietary groups (Fig. 4b) was performed by calculating specific band area ratios representative of the relative amounts of lipids (LIP/TBM), fatty acids (FA/TBM), proteins (PRT/TBM), carbohydrates (COH/TBM), phosphates (PH/TBM) and of the degree of unsaturation in lipid alkyl chains (CH/TBM) (see Supplementary Information for methods). Statistically significant lower amounts of total lipids (LIP/TBM, *p* < 0.05) and higher amounts of proteins (PRT/TBM, *p* < 0.05) were detected in Hi75 and Hi100 brain samples compared to the other experimental groups. A statistically significant decrement in unsaturated fatty acids (FA/TBM and CH/TBM, *p* < 0.05) and carbohydrates (COH/TBM, *p* < 0.05) was detected in all the dietary groups on BSF-based diets. No statistically significant differences were observed in all dietary groups as regards phosphate groups (PH/TBM).

#### Liver samples

**Figure 5:**
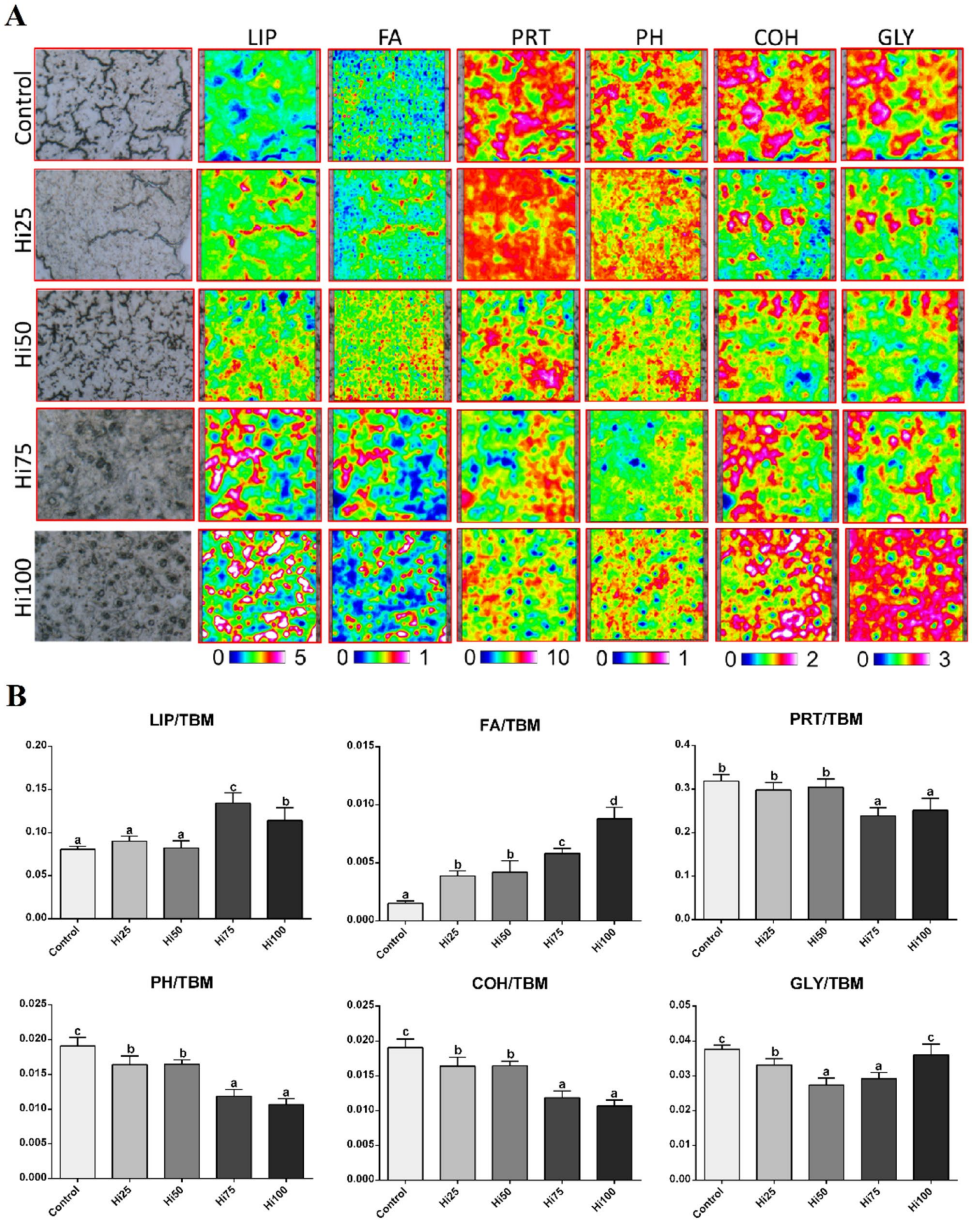
(**A**) Microphotographs, hyperspectral analysis, and (**B**) biochemical composition of representative liver sections belonging to all dietary groups. (**A**) False color images showing the topographical distribution of lipids (LIP), fatty acids (FA), proteins (PRT), phosphate groups (PH), carbohydrates (COH), and glycogen (GLY). (**B**) Univariate analysis of the following band area ratios: LIP/TBM (relative amount of total lipids); FA/TBM (relative amount of fatty acids); PRT/TBM (relative amount of total proteins); PH/TBM (relative amount of phosphate groups); COH/TBM (relative amount of total carbohydrates), and GLY/TBM (relative amount of glycogen). Data are reported as mean ± SD. Statistically significant differences are indicated by different letters (*p* < 0.05). Zebrafish fed diets including 0, 25, 50, 75 and 100% of BSF meal (Control, Hi25, Hi50, Hi75 and Hi100, respectively).

The hyperspectral imaging analysis of representative liver sections of zebrafish belonging to all dietary groups is showed in Fig. 5A. The generated false color images displayed the topographical distribution of lipids (LIP), fatty acids (FA), proteins (PRT), phosphates (PH), carbohydrates (COH) and glycogen (GLY). By considering the different scales adopted for each macromolecule, livers of Control group were characterized by a higher amount of proteins (PRT images, numerical scale 0–10) with respect to lipids (LIP images, numerical scale 0–5) and glycogen (GLY images, numerical scale 0–3). As regards the effects of the different diets, in all dietary groups containing increasing inclusion levels of BSF meal, the increment of total lipids (LIP images) and fatty acids (FA images) and the decrement of proteins (PRT images) and phosphate groups (PH images) were observed. Minor amounts of total carbohydrates and glycogen were also detected in Hi25, Hi50 and Hi75 dietary groups respect to Control, while Hi100 samples showed to have the highest levels.

The biochemical composition of liver samples of all dietary groups (Fig. 5B) was then investigated by the univariate analysis of specific band area ratios, representative of the relative amount of lipids (LIP/TBM), fatty acids (FA/TBM), proteins (PRT/TBM), phosphates (PH/TBM), carbohydrates (COH/TBM), and glycogen (GLY/TBM) (see Supplementary Materials for methods).

Higher amounts of total lipids (LIP/TBM, *p* < 0.05) and lower amounts of total proteins (PRT/TBM, *p* < 0.05) were detected only in Hi75 and Hi100 liver samples; an increasing trend in fatty acids amount (FA/TBM, *p* < 0.05) was detected in all livers samples of fish fed diets with increasing BSF meal inclusion levels, together with a decrease of both carbohydrates (COH/TBM, *p* < 0.05) and phosphates (PH/TBM, *p* < 0.05); lower levels of glycogen (GLY/TBM, *p* < 0.05) were found in Hi50 and Hi75 groups.

### Microbiome

After sequencing and quality filtering 51,554 reads were used for the downstream analysis with a median value of 10,572 ± 2411 reads/sample.

**Figure 6:**
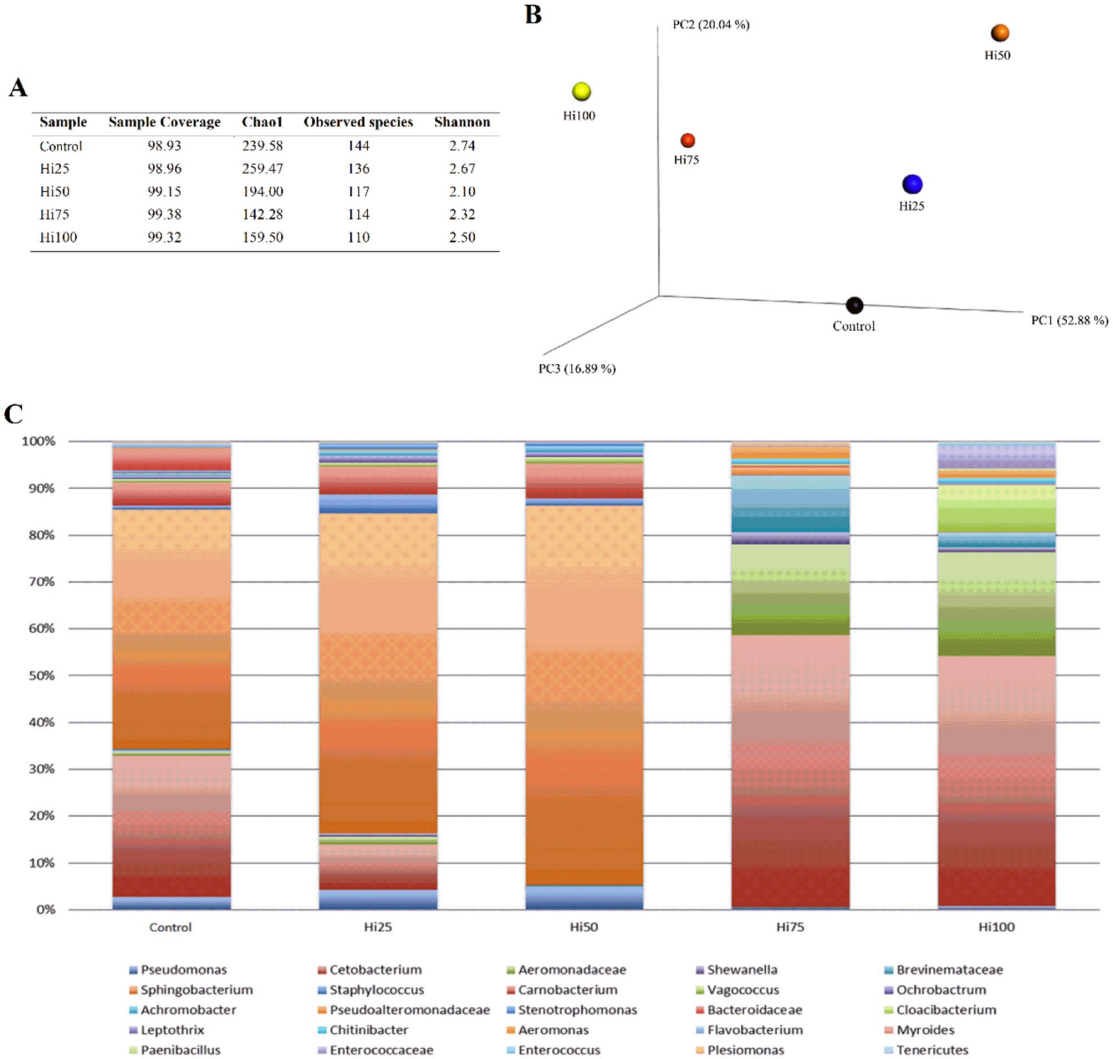
Microbiome of zebrafish fed diets including 0, 25, 50, 75 and 100% of BSF meal (Control, Hi25, Hi50, Hi75 and Hi100, respectively). (**A**) Number of sequences analysed, observed diversity and sample coverage for 16 s rrna amplicons acquired from zebrafish gut samples. (**B**) Principal coordinates analysis of weighted UniFrac distances for 16S rRNA gene sequence data assembled as a function of the dietary BSF amount. The first component (PC1) accounts for the 52.88% of the variance, the second one (PC2) for the 20.04% and the third one (PC3) for the 16.89%. (**C**) Frequency of the major taxonomic groups identified by sequencing. Only OTUs with an incidence > 0.2% are shown.

The estimated sample coverage indicated that there was a satisfactory coverage of all the samples (median value of 99%) and by comparing the alpha-diversity value a reduction both in number of species and in the chao1 index was correlated with the increase of BSF inclusion level in the diets (Fig. 6A). By plotting the Principal Coordinate Analysis (PCoA) of the UNIFRAC distance matrix (Fig. 6B) a separation of the samples as functions of the amount of BSF meal inclusion level in the diet was observed. Figure 6c displays the microbiota composition distribution across samples. In more detail, *Sphingobacterium* dominated the microbiota in Control, Hi25 and Hi50 fish, whereas the same genus was absent in Hi75 and Hi100 groups. In the Hi75 and Hi100 fish gut, *Cetobacterium*, Aeromonadaceae, and Brevinemataceae reached 50%, 20% and 10% of the relative abundance, respectively. The presence of *Cloacibacterium*, Enterococcaceae, Brevinemataceae, *Aeromonas* and *Shewanella* was also observed in Hi75 and Hi100 fish groups (Fig. 6C).

### Real-time PCR results

#### Growth factors

**Figure 7:**
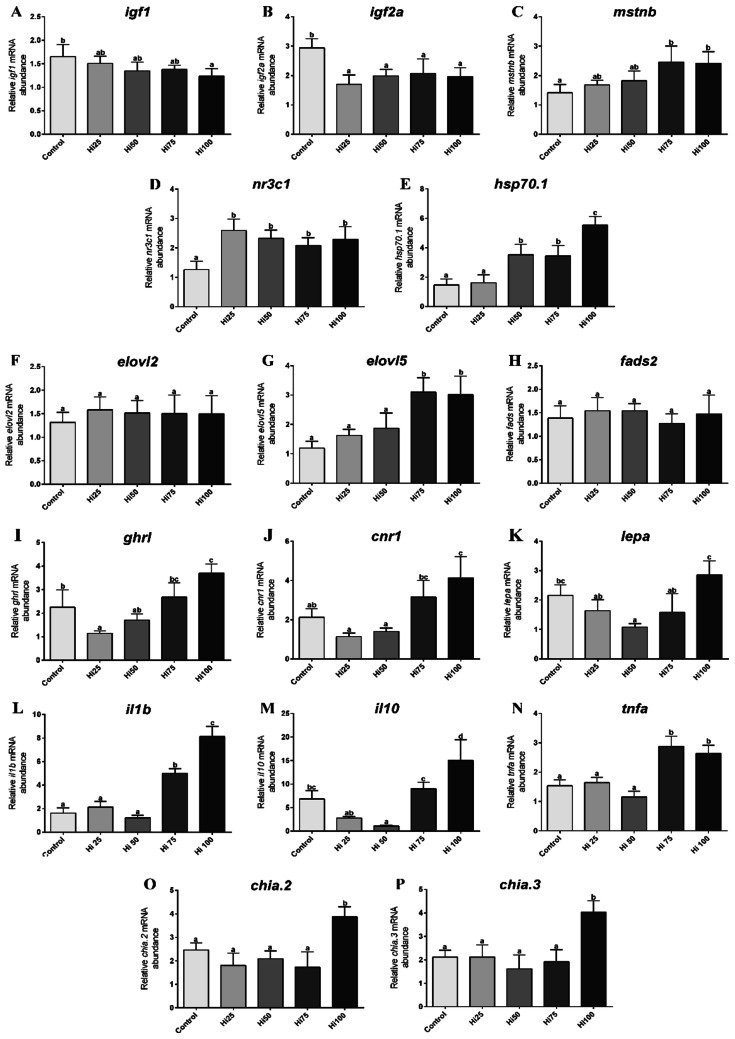
Relative mRNA abundance of genes analyzed in zebrafish fed on the different experimental diets (Control, Hi25, Hi50, Hi75 and Hi100). (**A**) *igf1*, (**B**) *igf2a*, (**C**) *mstnb,* (**D**) *nr3c1*, (**E**) *hsp70.1,* (**F**) *elovl2*, (**G**) *elovl5* and (**H**) *fads2* were analysed in liver samples; (**I**) *ghrl*, (**J**) *cnr1*, (**K**) *lepa,* (**L**) *il1b*, (**M**) *il10*, (**N**) *tnfa,* (**O**) *chia.2* and (**P**) *chia.3* were analysed in intestine samples. Different letters specify statistically significant differences among groups (*p* < 0.05). Values are showed as mean ± SD (n = 5).

As reported in Fig. 7A, insulin-like growth factor 1 (*igf1*) gene expression showed a slight downregulation (*p* < 0.05) from Control to Hi100. Considering insulin-like growth factor 2a (*igf2a*) gene expression (Fig. 7B), all the experimental groups fed on BSF-based diets were characterized by a significant (*p* < 0.05) downregulation with respect to Control. An opposite trend was observed for myostatin b (*mstnb*) gene expression (Fig. 7C) that was upregulated (*p* < 0.05) particularly when considering the Hi75 and Hi100 *vs* Control groups.

#### Stress response

As regards glucocorticoid receptor (nuclear receptor subfamily 3, group C, member 1; *nr3c1*) gene expression (Fig. 7D), all the experimental groups fed on BSF-based diets were characterized by a significant (*p* < 0.05) upregulation with respect to Control. Similarly, considering heat shock protein (*hsp70.1*) gene expression (Fig. 7E), Hi50, Hi75 and Hi100 groups showed a significant (*p* < 0.05) upregulation respect to Control and Hi25 group, which did not present significant differences between them.

#### Lipid metabolism

As concerns fatty acid elongase 5 (*elovl5*) gene expression (Fig. 7F), no significant differences were evident among Control, Hi25 and Hi50 groups, while Hi75 and Hi100 groups showed a significant (*p* < 0.05) upregulation respect to the other experimental groups.

Differently, considering fatty acid elongase 2 (*elovl2*) and fatty acid desaturase 2 (*fad*s*2*) gene expression (Fig. 7G,H) no significant differences were detected among the experimental groups.

#### Appetite

The experimental groups fed on BSF-based diets showed a significant (*p* < 0.05) dose-dependent increase in ghrelin (*ghrl*) gene expression (Fig. 7I). Control group presented a significant (*p* < 0.05) *ghrl* upregulation than Hi25 group and a significant *ghrl* downregulation than Hi100 group. Considering cannabinoid receptor 1 (*cnr1*) gene expression (Fig. 7J), no significant differences were detected among Control, Hi25 and Hi50 group. Differently, Hi75 and Hi100 groups showed a significant (*p* < 0.05) *cnr1* upregulation respect to the other groups (with the exception of Control and Hi75 group which did not evidence significant differences between them). Finally, as regards leptin a (*lepa*) gene expression (Fig. 7K), only Hi50 group was characterized by a significant (*p* < 0.05) downregulation with respect to Control. Furthermore, Hi100 group showed a significantly (*p* < 0.05) higher gene expression with respect to the other experimental groups fed on BSF-based diets.

#### Immune response

Considering genes involved in the immune response (interleukin 1β, interleukin 10, tumor necrosis factor a; Fig. 7L-N), groups fed the highest BSF inclusion levels (Hi75 and Hi100) showed a significant (*p* < 0.05) upregulation with respect to Control, Hi25 and Hi50 groups, while no significant differences (*p* < 0.05) were detected among them (with the exception of Hi50 group *il10* gene expression which showed a significant upregulation than Control).

#### Chitinases

Considering genes involved in the enzymatic hydrolysis of chitin (*chia.2* and *chia.3*), Hi100 group showed the highest gene expression (*p* < 0.05) with respect to all the other experimental groups which did not show significant differences among them (Fig. 7O,P).

### Behaviour

#### Open-field test

**Figure 8:**
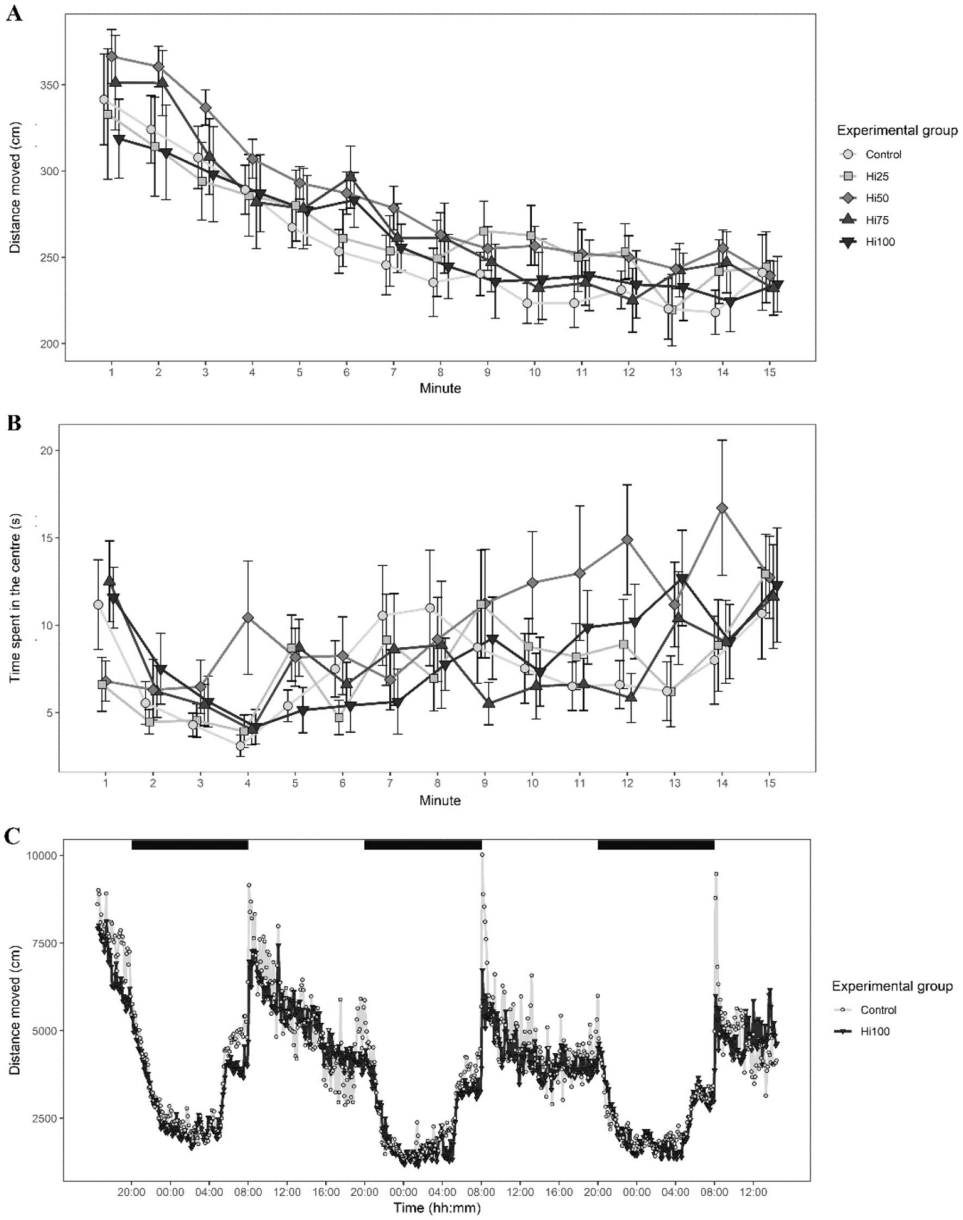
Behaviour**.** (**A**) activity and (**B**) time spent in the centre of the arena of zebrafish and tested in the open-field test. Dots represent means and error bars represent SE. Data are plotted in 1-min blocks. (**C**) Photic entrainment test: locomotor activity of zebrafish exposed to a 12 h:12 h light:dark cycle in the photic entrainment test. Black bars at the top of the graph indicate dark phases the LD cycles (light-on: 08:00, lights-off: 20:00). Dots represent means of 6-min time blocks. Zebrafish fed diets including 0, 25, 50, 75 and 100% of BSF meal (Control, Hi25, Hi50, Hi75 and Hi100).

Results of the open-field test are reported in Fig. 8A,B. Diets did not show a significant effect on fish behavior either considering the activity, measured as distance moved (ANOVA, F_4,75_ = 0.532, *p* = 0.713), or as the time spent in the centre of the arena (F_4,75_ = 0.689, *p* = 0.602). For both variables, the ANOVA test found a significant effect of time (activity: F_1,1119_ = 446.499; *p* < 0.001; time spent in the center: F_1,1119_ = 12.569; *p* < 0.001), indicating that fish changed their behavior over the time and therefore responded as expected to the open-field test.

#### Photic entrainment test

Because of the absence of a diet effect in the open field test, we investigated the behavioural photic entrainment only in two groups: Control and Hi100 (Fig. 8C). The ANOVA test evidenced that fish locomotor activity significantly varied between light and dark phase with the typical pattern of diurnal species (F_1,5590_ = 5,590.212, *p* < 0.001). However, the Control and Hi100 fish did not show significant behavioral alterations either considering the entire testing time (F_1,6_ = 0.008, *p* = 0.931) or the light and dark phases (F_1,5590_ = 0.275, *p* = 0.560).

## Discussion

In light of a sustainable circular economy, insects represent a very promising example of bio-converting organisms^7,9,19^. The substitution of dietary FM with BSF prepupae meal in aquafeeds is still controversial possibly due to differences in the diet formulation, the insects’ biomass nutritional quality and the fish species used during the trials^7,10,11^. Growth substrate selection plays a key role on the nutritional quality of BSF biomass^32^ and several studies have already demonstrated that BSF prepupae biomass can be improved by using an organic substrate containing proper amounts of PUFAs^6^. In particular, Truzzi et al. (2020)^9^ demonstrated that the addition of a 10% (w/w) of *Schizochytrium* sp. to the growth substrate was an efficient method to enrich BSF final biomass in terms of PUFAs and to increase, with respect to previous studies on zebrafish, the BSF meal inclusion level in the fish diets without affecting fish welfare^21,29,33^. However, the potential of these enriched BSF prepupae as alternative feed ingredient for fish was evaluated only during the fish larval stage^19^. In accord to previous studies^11,12,34^, the present results evidenced that fish growth was positively affected by the increasing inclusion levels of enriched BSF prepupae meal in the diets. Surprisingly, and differently from previous studies^21,29,33^, in the present research, biometric results were not fully supported by the molecular ones that evidenced a decreasing *igf1* and *igf2a* gene expression and an opposite trend for *mstnb* in the liver, followed by the increasing dietary BSF meal inclusion levels. It should be pointed out that the principal environmental regulator of the growth hormone (GH)-IGF axis is the organisms’ nutritional status^35^. Specifically, it is well established, that this axis can be differentially regulated by the dietary lipid content, at least at transcription level^36,37^. At this regard, Bertucci and collaborators (2019)^35^ reported that a large dietary replacement of highly unsaturated FA by saturated and monounsaturated ones caused a similar GH-IGFs pattern to that observed in starved fish. During fasting, both plasma Igfs concentration and liver *igfs* mRNA levels typically decline^38^, while GH plasma levels raise to limit growth and stimulate lipolysis^35^. This condition agrees with the higher intestine *ghrl* and *cnr1* gene expression observed in Hi75 and Hi100 zebrafish groups since the regulation of orexigenic signals (ghrelin in particular) is associated with different factors including GH and Igf1 concentrations^39^. Furthermore, as reported by previous studies on zebrafish, fish fed BSF-based diets showed better growth performances with respect to a control diet during the larval phase^19,33^, while this was not evident over a six-months feeding trial^21^. In the present study, a reduction in the SGR% difference among the experimental groups was evident (especially if compared to the larval stage^19^) and this can be related to the well-known different growth rates of larval, juvenile and adult fish^40^.

BSF-based diets used in this study showed a higher SFAs and a lower PUFAs content with respect to Control diet and their lipid profile affected zebrafish FA profile. However, differences among FAs classes were less evident in fish compared to those detected in diets because zebrafish, as a freshwater species, are able to synthesize HUFAs starting form shorter-chain precursors through the hepatic elongation and desaturation pathways^41^. In fact, in the present study, Hi75 and Hi100 groups showed higher liver *elovl5* gene expression, a transcript that codifies for the Elovl5 enzyme that is involved in the first step of HUFAs biosynthesis, compared to the other experimental groups^41^. Conversely, both *elovl2* and *fads2* did not show significant differences among experimental groups.

Furthermore, the role of chitin in aquafeeds is still controversial because high inclusion levels may induce a general reduction of diet digestibility and intestinal inflammation^14^. However, no signs of inflammation were detected by histological analyses in the intestine samples. These results are possibly related to the digestion of chitin through the activation of specific intestinal chitinases (*chia.2* and *chia.3* which were highly expressed in Hi100 group), the immunomodulatory role of ghrelin or by the anti-inflammatory, antibacterial and antiviral properties of medium-chain fatty acids (especially lauric acid, C12) which are particularly abundant in the BSF-based diets^19^. Furthermore, ghrelin (highly expressed in the intestine of Hi75 and Hi100 groups) also plays an important role in inflammatory responses, mainly through the regulation of cytokine production^42^ as confirmed by the molecular markers involved in the immune response analysed in intestine samples. Specifically, *il1b*, *il10* and *tnfa* analysed in intestine samples were significantly upregulated in Hi75 and Hi100 groups, compared to the other experimental groups.

Increasing inclusion levels of BSF meal in the diets are known to affect lipid accumulation in fish liver^13,21^. In particular, both histological and spectroscopic analyses detected a severe degree of hepatic steatosis in Hi75 and Hi100 zebrafish. This pathological condition (that caused an increase of stress markers gene expression)^43^ has already been related to a high SFA content and a high n6/n3 ratio in the diet^21^. Generally, fish require proper amounts of dietary PUFAs as they play an important role in the correct development of neural system^41^. Furthermore, altered dietary SFA and n6 intake have been related to behavioural and cognitive impairments in humans and rodents^26^. The FTIR analysis of brain samples evidenced that increasing inclusion levels of dietary BSF meal caused a drastic decrease of unsaturated fatty acids and carbohydrates in brain, mainly in Hi75 and Hi100 groups. Consequently, based on studies in rodents, we expected alterations of the fish behaviour^44,45^. Conversely, we did not observe effects of the diet in the behavioural tests suggesting that the different FA composition of brain tissues was not affecting fish behaviour. Further studies are necessary to address the link between fatty acid composition in the diet and fish behaviour.

Finally, high gut microbiota biodiversity is a desirable feature because it is usually associated with a healthy host^17^. However, it is well established that prolonged SFA and n6 consumption is able to reduce gut microbiota variability^27^. Specifically, Peng et al. (2019)^46^ reported a significantly change in the composition and nutrient metabolism of the intestinal microbial community of rice field eel (*Monopterus albus*) dependent on the dietary fat. Dietary lipids were able to modulate the microbial diversity and, in some cases, to stimulate massive proliferation of *Cetobacterium*, suggesting that dietary lipids could disturb the balance of intestinal microbiota.

In the same way, in the present study, increasing inclusion levels of BSF meal in the diets resulted in a reduction in microbiota biodiversity. This result is in contrast with previous studies reporting an increase in gut microbial richness and diversity with dietary administration of BSF meal^16^. However, in the present study, relative abundances of *Cetobacterium* increased in accordance with increasing BSF inclusion levels in the zebrafish diet. Even if a different diet, based on three graphene-family materials, was used, the results reported by Zheng et al. (2019)^47^ showed the influence of the diet on the occurrence of *Cetobacterium* in zebrafish gut. Indeed, a compensatory enrichment of the taxa might occur in the gut microbiota of fish in case of insufficient provision (or malabsorption) of cobalamin (vitamin B_12_) in the diet, being *Cetobacterium* able to synthesize such a vitamin without a dietary source^48^. Based on the available scientific literature and the results of the present study, the presence of *Cetobacterium* in the fish gut suggests a possible role for this bacterial genus in maintaining fish health and can be implied in maintaining brain’s function since vitamin B_12_ deficiency has been related to cognitive decline, and subsequent dementia^49^. Further research is needed to better clarify the dynamics and interactions between the host and this microbial genus.

In conclusion, while fish behaviour was not affected by the dietary treatments. results evidenced that Hi50 diet represented the best compromise between ingredient sustainability and proper fish growth and welfare. Fish fed with higher BSF inclusions (75 and 100%) showed hepatic steatosis, microbiota modification, higher lipid content, fatty acid modification and higher expression of immune response markers. Novel feed formulations (like those here presented) are ecologically sustainable and do not alter fish welfare, sustaining EU aquaculture priorities like productivity, sustainability and animal welfare. Over the last years the circular economy concept has become extremely important with EC Directive No. 2008/98 establishing the order of priority in the choice of waste management, the first being their reuse and the last being their landfill disposal. Insects represent excellent bio-converting organisms, which are able to convert land produced organic by-products in a valuable biological mass rich in proteins and lipids to be used in aquaculture. In this sense, the present results can sustain the aquaculture production by providing information on how to formulate sustainable practical diets in which unsustainable ingredients (FM) are substituted with sustainable ones (insect meal). By testing different BSF meal inclusion levels, the highest inclusion level (50%) which did not affect fish growth, quality and welfare was identified. Finally, by projecting these results at a global scale, a 50% substitution of FM with BSF meal represents an important goal for a more sustainable aquaculture industry.

## Methods

### Ethics

All procedures involving animals were conducted in line with the Italian legislation and approved by the Ethics Committee of Università Politecnica delle Marche and the Italian Ministry of Health (626/2018-PR).

### Insects rearing and fish diet production

Insects rearing and fish diet production were performed according to Zarantoniello et al. (2020)^20^. For more details, please see Supplementary Information section.

### Experimental design

Zebrafish larvae (information on zebrafish culture are reported in Supplementary Information section), were initially reared in fifteen 20L tanks to set up the five experimental dietary treatments; each experimental group was composed of 1500 larvae (n = 3). The water in the larval tanks had the same chemical-physical characteristics of the parent’s tank and was gently replaced 10 times a day by a dripping system^50^. After 30 days post spawning, fish of each tank were gently transferred in bigger tanks (80L; 15 in total, 3 per each dietary group) equipped with mechanical and biological filtration (Panaque, Capranica, VT, Italy). All the tanks were siphoned 30 min after feeding to remove possible feed excess and dead specimens. The required fish were sampled at 60 days post fertilization (dpf), euthanized with a lethal dose of MS222 (1 g/L) and properly stored for further analyses.

### Feeding schedule

The duration of the feeding trial was 57 days. Starting from 5 to 60 dpf, zebrafish were fed as follows: Control group: fish fed diet 0% insect meal (Control diet); fish fed the diet including 25% BSF full-fat prepupae meal (Hi25 diet); fish fed the diet including 50% of BSF full-fat prepupae meal (Hi50 diet); fish fed the diet including 75% BSF full-fat prepupae meal (Hi75 diet); fish fed the diet including 100% BSF full-fat prepupae meal (Hi100 diet). Feed particle sizes were < 100 µm from 5 to 15 dpf, 101–200 µm from 16 to 30 dpf and 201–400 µm from 31 to 60 dpf. Zebrafish were fed the experimental diets (2% body weight, BW) twice a day and, in addition, from 5 to 10 dpf, all groups were fed (one feeding in the morning) on the rotifer *Brachionus plicatilis* (5 ind/mL) according to Lawrence et al. (2012)^51^.

### Biometry

For growth measurements, 60 fish per dietary group (n = 3) were randomly collected from the different tanks at hatching (3dpf) and at the end of experiment (60dpf). At 3 dpf, wet weight was measured on pools of five larvae, while at 60dpf fish were individually measured. The wet weight was determined by an OHAUS Explorer (OHAUS Europe GmbH, Greifensee, Switzerland) analytical balance (precision: 0.1 mg). Specific growth rate (SGR) was calculated as follows: SGR% = [(lnW*f* − lnW*i*)/t) × 100, where W*f* is the final wet weight, W*i*, the initial wet weight, and t, the number of days (57). Survival was evaluated at the end of the experiment (60 dpf) by counting the number of fish respect to the initial larvae.

### Fatty acid composition

The experimental diets and fish deprived of the viscera (15 per dietary group; n = 3) were analyzed for fatty acid composition. Samples were minced and homogenized (homogenizer MZ 4,110, DCG Eltronic, Monza, Italy), and freeze-dried (Edwards EF4, Crawley, Sussex, England). Lipid extraction was carried out with the Folch method (1957)^52^ for experimental diets and with Microwave-Assisted Extraction (MAE) for fish^53^. All lipid extracts were evaporated under laminar flow inert gas (N_2_) until constant weight and re-suspended in 0.5 ml of n-epthane. Fatty acid methyl esters (FAMEs) were prepared according to Canonico et al. (2016)^54^ using methyl ester of nonadecanoic acid (19:0; Dr. Ehrenstorfer GmbH, Augsburg, Germany) as internal standard. FAMEs were determined by an Agilent-6890 GC System (Milano, Italy) coupled to an Agilent-5973 N quadrupole Mass Selective Detector (MS) (Milano, Italy). A CPS ANALITICA CC-wax-MS (30 m × 0.25 mm ID, 0.25 μm film thickness) capillary column was used to separate FAMEs. Instrumental conditions for the studied matrices were set up, according to Truzzi et al., 2018^55^. For each analysed sample, at least three runs were performed on the GCMS.

### Histology

Intestines and livers collected from 15 different fish specimens from dietary group (n = 3) were randomly collected and processed according to Cutrignelli et al. (2018)^56^. For details, please see Supplementary Information section. Moreover, to ascertain the degree of fat accumulation in liver, a quantitative analysis was performed on a significant number of histological sections from each experimental group in triplicate (n = 9). Not evaluable areas such as blood vessels were not considered. The percentage of fat fraction (PFF) was calculated by mean of the ImageJ software setting a homogeneous threshold value.

### FTIR measurements

Brains and livers from 15 fish specimens for dietary group (n = 3) were collected and quickly dissected and immediately frozen at –80 °C. Samples were then prepared for infrared spectroscopy (IR) measurements as reported in Supplementary Information section.

### Microbiome

#### RNA extraction and cDNA synthesis

Extracted intestines (60 per dietary treatment; n = 3) were added with sterile physiological solution (0.85% NaCl, w/v) at a 1:10 ratio and homogenized for 3 min at 260 rpm in Stomacher apparatus (400 Circulator, International PBI, Milan, Italy). An aliquot (500 µL) of each 10^−1^ dilution was centrifuged for 10 min at 14,000 rpm to produce cell pellets subsequently protected by RNA later Stabilization Solution (Ambion, Foster City, CA, USA) and stored at − 80 °C. Total microbial RNA was extracted from cell pellets by Quick-RNA Miniprep kit (Zymo Research, Irvine, CA, USA) following the manufacturer’s instructions. The extracted RNAs were checked for the quantity, purity and the absence of DNA contamination as previously described by Zarantoniello et al. (2020)^19^. SensiFAST cDNA Synthesis Kit for RT-qPCR (Bioline, London, UK) was used for the synthesis of the cDNA starting from 10 µL each sample RNA.

#### 16S rRNA amplicon target sequencing

V3-V4 region of the 16S rRNA gene was amplified via PCR as previously described by Klindworth et al. (2013)^57^ using cDNA from each sample as a template. PCR products were prepared for the sequencing by MiSeq Illumina instrument (Illumina) with V3 chemistry as previously detailed by Zarantoniello et al. (2020)^19^. Paired-end reads were assembled with FLASH^58^ and quality filtered (at Phred < Q20) using QIIME 1.9.0 software and the pipeline recently described^59^. OTUs were clustered at 97% of similarity and taxonomy was assessed by Greengenes database v. 2013. OTU table was rarefied at the lowest number of sequence and display the higher taxonomy resolution.

### Molecular analyses

#### RNA extraction, cDNA synthesis and Real-Time PCR

Total RNA extraction, cDNA synthesis and Real-Time PCR from both liver and intestine samples from 15 different specimens from dietary group (n = 3) were performed according to Piccinetti et al. (2013)^60^. For details on methods and primers sequences (reported in Supplementary Table S3), please see Supplementary Information section.

### Behavioural tests

#### Open-field test

Our open-field test followed the procedure commonly adopted to study fish^61^ and to assess effects of diets^62^. Each individual (n = 16 zebrafish per dietary group; 80 zebrafish overall) was placed in an unfamiliar, empty arena (40 × 40 cm, 10 cm of water) for a brief period (15 min). A light source [Light-emitting diodes (LED), warm white; Superlight Technology Co. Ltd., Shenzhen, China] was placed 1 m above the area. The spontaneous behaviour of the subject was recorded by means of videotracking system equipped with an IR-sensitive camera (Monochrome GigE camera, Basler, Ahrensburg, Germany; resolution: 1,280 × 1,024). Using Ethovision 11 software (Noldus Information Technology, Wageningen, NL), two variables were analysed: the activity of the subject (measured as distance travelled) and the time spent in the centre the open field (1 body length from the edges). Bolder, more explorative, and less anxious fish were expected to travel greater distances in the open field and spend more time in the central area.

#### Photic behavioural entrainment test

The photic behavioural entrainment test was performed following a standard procedure^63^. Because the test required extended recordings, zebrafish were tested in groups formed by four individuals to avoid social isolation. The subjects were different from those used in the previous behavioural test, and therefore data of the two tests were independent. Because the previous behavioural test did not provide evidence of different behaviours between the dietary groups, here only fish from the Control group (n groups = 4, n zebrafish = 16) and from the dietary treatment with higher insect meal inclusion (Hi100 diet; n groups = 4, n zebrafish = 16) were compared. Each group was kept in a square arena (20 × 20 cm) and exposed to a 12:12 light–dark cycle. For light sources, LED (Superlight Technology Co. Ltd., Shenzhen, China) were used. Irradiance was measured with a radiometer (DO9721, Probe LP9021 RAD, Spectral range 400–950 nm, DeltaOHM, Padova, Italy) and set at 0.6 W/m2. The temperature was held constant at 28 °C by means of a thermostatically controlled heater. Zebrafish locomotor activity was recorded for 70 consecutive hours and then analysed by Ethovision 11. The IR-sensitive camera was set to 5 frames per second. Locomotor activity of each group was calculated as the total distance moved during a 6 min time window (700 observations per subject). A minimal distance moved of 2 mm was used. In case of normal photic behavioural entrainment, zebrafish were expected to show markedly diurnal activity^63^.

### Statistical analysis

All data (except for microbiome) were analyzed by one-way ANOVA, with diet as the explanatory variable. All ANOVA tests were followed by Tukey’s post-hoc test. The statistical software package Prism5 (GraphPad Software) was used. Significance was set at *p* < 0.05 and all the results are presented as mean ± SD. As regards microbiome, bioinformatics analysis were performed as described by Osimani et al. (2019)^18^ and Zarantoniello et al. (2020)^19^.

## Supplementary information

Supplementary information
