# Supplementary material for: Zebrafish (Danio rerio) physiological and behavioural responses to insect-based diets: a multidisciplinary approach
Source: Sci Rep. 2020 Jun 30;10:10648. doi: 10.1038/s41598-020-67740-w (PMC7326965; doi:10.1038/s41598-020-67740-w)
Supplement: Supplementary file 1 — Supplementary information [file 41598_2020_67740_MOESM1_ESM.docx]

**SUPPLEMENTARY INFORMATION**

**Zebrafish (*Danio rerio*) physiological and behavioural responses to insect-based diets: a multidisciplinary approach**

Matteo Zarantoniello^1^, Basilio Randazzo^1^, Giorgia Gioacchini^1^, Cristina Truzzi^1^, Elisabetta Giorgini^1^, Paola Riolo^2^, Giorgia Gioia^1^, Cristiano Bertolucci^3^, Andrea Osimani^2^, Gloriana Cardinaletti^4^, Tyrone Lucon-Xiccato^3^, Vesna Milanović^2^, Anna Annibaldi^1^, Francesca Tulli^4^, Valentina Notarstefano^1^, Sara Ruschioni^2^, Francesca Clementi^2^ & Ike Olivotto^1*^

^1^ Dipartimento di Scienze della Vita e dell’Ambiente, Università Politecnica delle Marche, via Brecce Bianche, 60131Ancona, Italy.

^2^ Dipartimento di Scienze Agrarie, Alimentari ed Ambientali, Università Politecnica delle Marche, via Brecce Bianche, 60131 Ancona, Italy.

^3^ Dipartimento di Scienze della Vita e Biotecnologie, Università di Ferrara, via L. Borsari 46, 44121 Ferrara, Italy.

^4^ Dipartimento di Scienze Agro-Alimentari, Ambientali e Animali (Di4A), Università di Udine, via Sondrio 2/A, 33100 Udine, Italy.

* i.olivotto@univpm.it

**Insect rearing**

The insect diet was formulated including a 10% (w/w) of *Schizochytrium* sp. to the coffee by-product (for details, please see Truzzi et al., 2020^9^ and Zarantoniello et al., 2020^20^). The freeze-dried *Schizochytrium* sp. was provided by AlghItaly Società Agricola S.R.L. (Sommacampagna, VR, Italy). Distilled water was added to feeding substrate to reach a moisture of ~70%.

The insect rearing was carried out in a climatic chamber at a 27±1 °C temperature, relative humidity of 65±5%, in continuous darkness. Six days old larvae [purchased from Smart Bugs s.s. Ponzano Veneto (TV), Italy] were hand counted and divided in groups of 640 larvae per replicate (n=65) for a total of 41,600 specimens. Each replicate consisted of a plastic box (57x38x16cm) screened with fine‐mesh cotton gauze and covered with a lid provided with 90 ventilation holes of 0.05cm Ø. Each larva was provided with a feeding rate of 100 mg/day. Food was prepared and added once a week (448 g for each box). Insects were visually inspected every day and when prepupae were identiﬁed by the change in tegument colour from white to black, they were collected, washed, dried and stored at -80 °C.

**Fish diets production**

Full-fat BSF prepupae were freeze dried, grinded with Retsch Centrifugal Grinding Mill ZM 1000 (Retsch GmbH, Haan, Germany) and used to prepare the experimental diets. A control diet (Hi0) containing FM, wheat gluten, pea protein concentrates and FO as major ingredients, was prepared according to a commercially available standard diet for zebrafish (Zebrafeed, Sparos ltd, Olhão, Portugal). The experimental diets were isonitrogenous (50%) and isolipidic (13%). Insect-based diets were prepared by including graded levels of insect meal (25%, 50%, 75%, and 100%, referred to as Hi25 and Hi50, Hi75 and Hi100, respectively) in the Hi0 formulation. The obtained diets were then grinded and stored in under vacuum bags at −20 °C until used. Feed samples were analyzed for moisture (AOAC #950.46), crude protein, CP (AOAC #976.05), ash (AOAC #920.153) and ether extract (EE; AOAC #991.36) contents according to AOAC 2006^63^.

The total lipid fraction of the test diets was extracted using chloroform-methanol (2:1 v:v) (Merck KGaA, Darmstadt, Germany) mixture according to Folch method (1957)^52^. Diet formulation and proximate composition are shown in Supplementary Table S1.

SUPPLEMENTARY TABLE S1. INGREDIENTS (g Kg^−1^) AND PROXIMATE COMPOSITION (g 100 g^−1^) OF THE EXPERIMENTAL DIETS USED IN THIS STUDY.

|  | **Hi0 (Control)** | **Hi25** | **Hi50** | **Hi75** | **Hi100** |
| --- | --- | --- | --- | --- | --- |
| ***Ingredients (g/kg)*** |  |  |  |  |  |
| Fish meal ^1^ | 470 | 400 | 250 | 110 | - |
| Vegetable mix ^2^ | 220 | 230 | 298 | 385 | 440 |
| Hi meal | - | 150 | 275 | 350 | 460 |
| Wheat flour ^3^ | 198 | 172 | 120 | 110 | 72 |
| Fish oil | 80 | 51 | 25 | 10 | - |
| Soy lecithin | 8 | 8 | 8 | 11 | 4 |
| Mineral and Vitamin supplements ^$^ | 14 | 14 | 14 | 14 | 14 |
| Binder | 10 | 10 | 10 | 10 | 10 |
| ***Proximate composition (%)*** |  |  |  |  |  |
| Dry Matter | 97.08 ± 0.06 | 95.78 ± 0.13 | 94.93 ± 0.05 | 93.63 ± 0.05 | 92.70 ± 0.04 |
| Crude protein, CP | 51.57 ± 0.13 | 50.75 ± 2.57 | 50.39 ± 0.28 | 51.23 ± 1.49 | 50.50 ± 3.15 |
| Ether extract, EE | 14.38 ± 0.64 | 13.10 ± 0.42 | 12.93 ± 0.38 | 13.24 ± 0.46 | 12.99 ± 0.51 |
| NFE | 21.32 ± 0.34 | 20.82 ± 1.00 | 20.64 ± 0.55 | 19.03 ± 0.67 | 18.47 ± 1.26 |
| Ash | 9.81 ± 0.25 | 11.11± 0.01 | 10.97 ± 0.00 | 10.13 ± 0.06 | 10.74 ± 0.13 |

^1^ Raw ingredients kindly supplyed by Skretting Italia; ^2^ Vegetable mix (pea protein concentrate : wheat gluten, 0.7:1 w/w) Lombarda trading srl, Casalbuttano & Uniti (CR, Italy) and Sacchetto spa (Torino, Italy); ^3^ Consorzio Agrario (Pordenone, Italy); ^$^ Mineral and Vitamin supplement composition (% mix): CaHPO_4_.2H_2_O, 78.9; MgO, 2.725; KCl, 0.005; NaCl, 17.65; FeCO_3_, 0.335; ZnSO_4_.H_2_O, 0.197; MnSO_4_.H_2_O, 0.094; CuSO_4_.5H_2_O, 0.027; Na_2_SeO_3_, 0.067; thiamine hydrochloride (vitamin B1), 0.16; riboflavin (vitamin B2), 0.39; pyridoxine hydrocloride (vitamin B6), 0.21; cyanocobalamine (vitamin B12), 0.21; niacin (vitamin PP or B3), 2.12; calcium pantotenate, 0.63; folic acid, 0.10; biotin (vitamin H), 1.05; myo-inositol (vitamin B7), 3.15; stay C Roche (vitamin C), 4.51; tocopherol (vitamin E), 3.15; menadione (vitamin K3), 0.24; retinol (vitamin A 2500 UI kg^−1^ diet), 0.026; cholecalciferol (vitamin D3 2400 UI kg^−1^ diet), 0.05; choline chloride, 83.99; ***** Values reported as mean of triplicate analyses; ^6^ n.d.: not determined

**Fish**

Zebrafish AB embryos were maintained 48h in a Tecniplast system (Varese, Italy), subjected to the following conditions: 28 °C temperature, pH 7.0, NO_2_ and NH_3_ concentrations < 0.01 mg/L, NO_3_ concentration < 10 mg/L, and photoperiod 12L/12D. After this first period, embryos were gently collected, counted under a stereomicroscope (Leica Wild M3B, Leica Microsystems, Nussloch, Germany) and randomly divided in five experimental groups (in triplicate) according to the five test diets.

**Fatty acid composition**

The zebrafish FAs composition is reported in Supplementary Table S2.

SUPPLEMENTARY TABLE S2. ZEBRAFISH FATTY ACID COMPOSITION (AS % OF TOTAL FAS).

|  | **Zebrafish** | | | | |
| --- | --- | --- | --- | --- | --- |
|  | Control | Hi25 | Hi50 | Hi75 | Hi100 |
| 10:0 | 0.02±0.01 | 0.04±0.01 | 0.12±0.01 | 0.13±0.01 | 0.15±0.02 |
| 12:0 | 0.27±0.01^a^ | 3.0±0.3^b^ | 6.2±0.2^c^ | 7.2±0.3^d^ | 8.3±0.5^e^ |
| 13:0 | 0.04±0.01 | 0.05±0.01 | 0.07±0.01 | 0.08±0.02 | 0.08±0.02 |
| 14:0 | 4.3±0.3^a^ | 4.5±0.3^a^ | 5.1±0.3^bc^ | 4.9±0.1^b^ | 5.2±0.4^c^ |
| 14:1n5 | 0.27±0.02 | 0.33±0.05 | 0.40±0.03 | 0.45±0.07 | 0.59±0.09 |
| 15:0 | 0.78±0.03 | 0.79±0.02 | 0.81±0.06 | 0.84±0.13 | 0.84±0.16 |
| 16:0 | 16.8±1.5^b^ | 15.9±0.3^a^ | 16.9±0.9^b^ | 17.9±1.1^c^ | 18.2±0.8^c^ |
| 16:1n9 | 0.76±0.05 | 0.78±0.03 | 0.90±0.17 | 0.89±0.12 | 0.87±0.07 |
| 16:1n7 | 7.7±0.2^b^ | 7.8±0.3^b^ | 7.8±0.1^b^ | 7.2±0.3^a^ | 7.1±0.1^a^ |
| 17:0 | 0.81±0.03 | 0.84±0.1 | 0.75±0.1 | 0.81±0.1 | 0.90±0.1 |
| 17:1n7 | 0.81±0.12^a^ | 1.1±0.04^b^ | 1.2±0.04^c^ | 1.4±0.11^d^ | 1.6±0.02^e^ |
| 18:0 | 4.8±0.8^a^ | 4.7±0.5^a^ | 5.0±1.0^a^ | 4.7±0.6^a^ | 5.4±1.1^a^ |
| 18:1n9 | 14.6±0.8^a^ | 14.7±0.5^a^ | 15.1±0.1^a^ | 15.3±0.9^a^ | 15.4±1.4^a^ |
| 18:1n7 | 3.4±0.2^c^ | 3.5±0.2^c^ | 3.0±0.2^b^ | 2.7±0.1^a^ | 2.9±0.1^b^ |
| 18:2n6 | 10.7±0.6^a^ | 11.3±0.2^b^ | 12.9±0.1^c^ | 15.7±1.0^e^ | 15.2±0.6^d^ |
| 18:3n6 | 0.33±0.02 | 0.38±0.02 | 0.40±0.04 | 0.58±0.06 | 0.52±0.03 |
| 18:3n3 | 2.2±0.2^c^ | 2.2±0.1^c^ | 2.0±0.2^ab^ | 2.1±0.3^bc^ | 1.8±0.2^a^ |
| 20:0 | 0.18±0.01 | 0.16±0.01 | 0.18±0.03 | 0.19±0.02 | 0.18±0.04 |
| 20:1n9 | 1.0±0.04^e^ | 0.83±0.06^d^ | 0.56±0.03^c^ | 0.43±0.05^b^ | 0.33±0.02^a^ |
| 20:2n6 | 0.32±0.03 | 0.32±0.02 | 0.32±0.05 | 0.32±0.01 | 0.44±0.06 |
| 20:3n6 | 0.38±0.04^a^ | 0.50±0.02^b^ | 0.68±0.03^c^ | 0.85±0.10^d^ | 1.2±0.1^e^ |
| 20:4n6 | 1.2±0.1^a^ | 1.5±0.1^b^ | 1.6±0.1^b^ | 1.8±0.1^c^ | 2.2±0.1^d^ |
| 20:3n3 | 0.18±0.02 | 0.16±0.01 | 0.13±0.02 | 0.11±0.02 | 0.13±0.04 |
| 20:5n3 | 10.7±0.7^e^ | 9.5±0.8^d^ | 6.1±0.8^c^ | 3.6±0.4^b^ | 2.0±0.5^a^ |
| 22:1n9 | 0.51±0.1 | 0.36±0.02 | 0.18±0.01 | 0.11±0.04 | 0.03±0.01 |
| 22:6n3 | 16.8±1.8^e^ | 14.7±1.1^d^ | 11.5±0.8^c^ | 10.0±0.2^b^ | 8.4±0.7^a^ |
| 24:1n9 | 0.21±0.03 | 0.13±0.01 | 0.04±0.01 | 0.02±0.01 | 0.01±0.01 |
| DHA/EPA | 1.6±0.1^a^ | 1.6±0.1^a^ | 1.9±0.1^b^ | 2.8±0.3^c^ | 4.4±0.4^d^ |

Zebrafish fed diets including 0, 25, 50, 75 and 100% of BSF meal (Control, Hi25, Hi50, Hi75 and Hi100). Means within rows bearing different letters are significantly different (p<0.05). Statistical analysis was performed only for FAs > 1%. FAs with a percentage <1% were excluded from any statistical analyses because their concentrations were close to the limit of detection.

**Histology**

Samples were fixed by immersion in Bouin’s solution (Sigma-Aldrich, Milano, Italy) and then stored at 4°C for 24h. Samples were washed three times with ethanol (70%) for ten minutes and preserved in the same ethanol solution. Samples were then dehydrated in crescent ethanol solutions (80, 95 and 100%), washed with xylene (Bio-Optica, Milano, Italy) and embedded in paraffin (Bio-Optica). Solidified paraffin blocks were cut with a microtome (Leica RM2125 RTS, Nussloch, Germany) and 5 µm sections were stained with Mayer hematoxylin and eosin Y (Sigma-Aldrich, Milano, Italy). Sections were observed using a Zeiss Axio Imager.A2 (Oberkochen, Germany) microscope in order to study the hepatic parenchyma and intestine morphology. Images were acquired by mean of a combined color digital camera Axiocam 503 (Zeiss, Oberkochen, Germany).

**FTIR measurements**

**Brain samples**. FTIR measurements of brain samples were carried out by using a Perkin Elmer Spectrum GX1 spectrometer (Waltham, Massachusetts, USA). Brain samples of each dietary group were pooled, minced, homogenized (homogenizer MZ 4110, DCG Eltronic, Monza, Italy) and freeze-dried (Edwards EF4, Crawley, Sussex, England). Then, homogenized samples were deposited onto the crystal of the U-ATR accessory for the analysis in reflectance mode. For each sample, 10 spectra were collected in the 4000-650 cm^-1^ spectral range with a spectral resolution of 4 cm^-1^; each spectrum was the result of 64 scans. Before each sample acquisition, a background spectrum was collected on the empty crystal. Raw IR spectra were converted in absorbance and corrected for the contribution of atmospheric carbon dioxide and water vapor (OPUS 7.1 software package, Bruker Optics GmbH, Ettlingen, Germany).

On these pre-processed spectra, the integrated areas of the following spectral ranges were determined: 3040-3000 cm^-1^ (=CH groups in lipid alkyl chains, CH), 3000-2800 cm^-1^ (CH2 and CH3 groups in lipid alkyl chains, LIP), 1772-1713 cm^-1^ (C=O moiety in fatty acids, FA), 1708-1480 cm^-1^ (Amide I and II bands of proteins, PRT), 1281-1191 cm^-1^ (phosphates, PH); ), 1191-1134 cm^-1^ (COH groups in carbohydrates, COH), 1134-996 cm^-1^ (phosphates and carbohydrates, PH-CARBO), 996-943 cm^-1^ (A-DNA), and 943-900 cm^-1^ (Z-DNA). The following band area ratios were then calculated: LIP/TBM, CH/TBM, FA/TBM, PRT/TBM, PH/TBM, COH/TBM, PH-CARBO/TBM, A-DNA/TBM, and Z-DNA/TBM. Each value was the ratio between the integrated area of the above defined spectral intervals and the integrated area of the whole spectrum (TBM).

For each dietary group, the average absorbance spectrum was also calculated together with the corresponding standard deviation spectra (average absorbance spectrum ± standard deviation spectra).

**Liver samples**. From the middle part of each liver sample, three thin sections (10 µm thick) were cut at 100 µm intervals by using a cryostat MC400 (Histo-Line Laboratories, Pantigliate, Milano, Italy) and deposited onto CaF_2_ optical windows (1 mm thick, 13 mm diameter). FTIR measurements were carried out by using a Bruker Invenio interferometer coupled with a Hyperion 3000 Vis-IR microscope and a bidimensional Focal Plane Array (FPA) detector (Bruker Optics GmbH, Ettlingen, Germany). On each section, 2/3 IR maps were collected in transmission mode in the 4000-800 cm^-1^ spectral region; each IR map was a square of 164 µm per side, and was formed by 4096 pixel/spectra (2.56x2.56 µm spatial resolution, 4 cm^-1^ spectral resolution, 128 scans). Before the acquisition of each IR map, a background spectrum was collected on a clean area of the CaF_2_ optical window. Raw IR maps were corrected to avoid the contribution of atmospheric carbon dioxide and water vapor and then vector normalized to correct small differences in the thickness of the sample (OPUS 7.1 software package, Bruker Optics GmbH, Ettlingen, Germany).

From each IR map, false color images representing the topographical distribution of specific biocomponents (such as lipids, fatty acids, proteins, etc.) were generated by integration under the following spectral ranges: 3000-2825 cm^-1^ (representative of lipids, LIP images), 1760-1725 cm^-1^ (representative of fatty acids, FA images), 1725-1480 cm^-1^ (representative of proteins, PRT images), 1280-1183 cm^-1^ (representative of phosphates, PH images), 1183-1136 cm^-1^ (representative of carbohydrates, COH images), and 1067-983 cm^-1^ (representative of glycogen, GLY images).

The univariate analysis of the biochemical composition of liver samples of all dietary groups was performed by calculating specific band area ratios representative of the relative amount of lipids (LIP/TBM), fatty acids (FA/TBM), proteins (PRT/TBM), phosphates (PH/TBM), carbohydrates (COH/TBM), and glycogen (GLY/TBM). Each value was the ratio between the integrated area of the above defined spectral intervals and the integrated area of the whole spectrum (TBM).

**Molecular analyses**

**RNA extraction and cDNA synthesis.** Total RNA extraction from both liver and intestine samples from 5 different specimens from each tank (15 per dietary group) were performed optimized using RNAzol RT reagent (Sigma-Aldrich, R4533) following the manufacturer’s instructions. Total RNA extracted was eluted in 40 µl of RNase-free water (Qiagen). Final RNA concentration was determined by the NanoPhotometer P-Class (Implen, München, Germany). RNA integrity was verified by GelRed^TM^ staining of 28S and 18S ribosomal RNA bands on 1% agarose gel. RNA was stored at -80°C until use. Finally, 1 µg of total RNA were used for cDNA synthesis, employing the LunaScript RT SuperMix Kit (New England Biolabs, Ipswich, Massachusetts, USA) following the manufacturer's instructions.

**Real-Time PCR.** PCRs were performed with SYBER green method in an iQ5 iCycler thermal cycler (Bio-Rad Laboratories) following Zarantoniello et al. (2020)^20^. Relative quantification of the expression of genes involved in fish growth (*igf1*, *igf2a* and *mstnb*), stress response (*nr3c1* and *hsp70.1*), long-chain polyunsaturated fatty acids biosynthesis (*elovl2*, *elovl5* and *fads2*), appetite response (*ghrl, cnr1* and *lepa*), immune response (*il1b*, *il10* and *tnfa*) and enzymatic hydrolysis of chitin (*chia.2* and *chia.3*) was performed. Actin related protein 2/3 complex, subunit 1A (*arpc1a*) and ribosomal protein, large, 13 (*rpl13*) were used as internal standards in each sample in order to standardize the results by eliminating variation in mRNA and cDNA quantity and quality. No amplification products were observed in negative controls and no primer-dimer formations were observed in the control templates. Amplification products were sequenced, and homology was verified. The data obtained were analysed using the iQ5 optical system software version 2.0 (Bio-Rad) including GeneEx Macro iQ5 Conversion and genex Macro iQ5 files. Primer sequences were designed using Primer3 (210 v. 0.4.0) starting from zebrafish sequences available in ZFIN. Primer sequences used were reported in Supplementary Table S3.

SUPPLEMENTARY TABLE S3. PRIMER SEQUENCES AND THE ZEBRAFISH INFORMATION NETWORK (ZFIN) USED IN THIS STUDY

| *Gene* | *Forward primer (5'- 3')* | *Reverse primer (5'- 3')* | *ZFIN ID* |
| --- | --- | --- | --- |
| *igf1* | 5'-GGCAAATCTCCACGATCTCTAC-3' | 5'-CGGTTTCTCTTGTCTCTCTCAG-3' | ZDB-GENE-010607-2 |
| *igf2a* | 5'-GAGTCCCATCCATTCTGTTG-3' | 5'-GTGGATTGGGGTTTGATGTG-3' | ZDB-GENE-991111-3 |
| *mstnb* | 5'-GGACTGGACTGCGATGAG-3' | 5'-GATGGGTGTGGGGATACTTC-3' | ZDB-GENE-990415-165 |
| *nr3c1* | 5'-AGACCTTGGTCCCCTTCACT-3' | 5'-CGCCTTTAATCATGGGAGAA-3' | ZDB-GENE-050522-503 |
| *hsp70.1* | 5'-TGTTCAGTTCTCTGCCGTTG-3' | 5'-AAAGCACTGAGGGACGCTAA-3' | ZDB-GENE-990415-91 |
| *elovl2* | 5'-CACTGGACGAAGTTGGTGAA-3' | 5'-GTTGAGGACACACCACCAGA-3' | ZDB-GENE-060421-5612 |
| *elovl5* | 5'-TGGATGGGACCGAAATACAT-3' | 5'-GTCTCCTCCACTGTGGGTGT-3' | ZDB-GENE-040407-2 |
| *fads2* | 5'-CATCACGCTAAACCCAACA-3' | 5'-GGGAGGACCAATGAAGAAGA-3' | ZDB-GENE-011212-1 |
| *ghrl* | 5'-CAGCATGTTTCTGCTCCTGTG-3' | 5'TCTTCTGCCCACTCTTGGTG-3' | ZDB-GENE-070622-2 |
| *cnr1* | 5'-AGCAAAAGGAGCAACAGGCA-3' | 5'GTTGGTCTGGTACTTTCACTTGAC-3' | ZDB-GENE-040312-3 |
| *lepa* | 5'-CTCCAGTGACGAAGGCAACTT-3' | 5'GGGAAGGAGCCGGAAATGT-3' | ZDB-GENE-081001-1 |
| *il1b* | 5'-GCTGGGGATGTGGACTTC-3' | 5'-GTGGATTGGGGTTTGATGTG-3' | ZDB-GENE-040702-2 |
| *il10* | 5'-ATTTGTGGAGGGCTTTCCTT-3' | 5'AGAGCTGTTGGCAGAATGGT-3' | ZDB-GENE-051111-1 |
| *tnfα* | 5'-TTGTGGTGGGGTTTGATG-3' | 5'-TTGGGGCATTTTATTTTGTAAG-3' | ZDB-GENE-050317-1 |
| *chia.2* | 5'-GGTGCTCTGCCACCTTGCCTT-3' | 5'-GGCATGGTTGATCATGGCGAAAGC-3' | ZDB-GENE-040426-2014 |
| *chia.3* | 5'-TCGACCCTTACCTTTGCACACACCT-3' | 5'-ACACCATGATGGAGAACTGTGCCGA-3' | ZDB-GENE-040426-2891 |
| *arpc1a* | 5'-CTGAACATCTCGCCCTTCTC-3' | 5'-TAGCCGATCTGCAGACACAC-3' | ZDB-GENE-040116-1 |
| *rpl13* | 5'-TCTGGAGGACTGTAAGAGGTATGC-3' | 5'-AGACGCACAATCTTGAGAGCAG-3' | ZDB-GENE-031007-1 |
